# Multistep Protein Unfolding Scenarios from the Rupture of a Complex Metal Cluster Cd_3_S_9_

**DOI:** 10.1038/s41598-019-47004-y

**Published:** 2019-07-19

**Authors:** Guodong Yuan, Qun Ma, Tao Wu, Mengdi Wang, Xi Li, Jinglin Zuo, Peng Zheng

**Affiliations:** State Key Laboratory of Coordination Chemistry, School of Chemistry and Chemical Engineering, Nanjing University, Nanjing, Jiangsu 21002 China

**Keywords:** Biophysical chemistry, Biophysics

## Abstract

Protein (un)folding is a complex and essential process. With the rapid development of single-molecule techniques, we can detect multiple and transient proteins (un)folding pathways/intermediates. However, the observation of multiple multistep (>2) unfolding scenarios for a single protein domain remains limited. Here, we chose metalloprotein with relatively stable and multiple metal-ligand coordination bonds as a system for such a purpose. Using AFM-based single-molecule force spectroscopy (SMFS), we successfully demonstrated the complex and multistep protein unfolding scenarios of the β-domain of a human protein metallothionein-3 (MT). MT is a protein of ~60 amino acids (aa) in length with 20 cysteines for various metal binding, and the β-domain (βMT) is of ~30 aa with an M_3_S_9_ metal cluster. We detected four different types of three-step protein unfolding scenarios from the Cd-βMT, which can be possibly explained by the rupture of Cd-S bonds in the complex Cd_3_S_9_ metal cluster. In addition, complex unfolding scenarios with four rupture peaks were observed. The Cd-S bonds ruptured in both single bond and multiple bonds modes. Our results provide not only evidence for multistep protein unfolding phenomena but also reveal unique properties of metalloprotein system using single-molecule AFM.

## Introduction

Proteins are the fundamental building blocks of life, and their correctly folded three-dimensional structure is essential for proper function after biosynthesis^1^. However, the protein folding and unfolding mechanism at the single-bond resolution remain a scientific challenge, which is difficult to characterize in detail. With the development of single-molecule techniques, it is now experimentally proved that most single-domain protein molecules (un)fold through a series of intermediates, eventually reaching fully folded or unfolded states^2–7^. However, most sequential unfolding scenarios observed are two-step with one or two different pathways, and multiple multistep (>2) unfolding scenario is reported in limited cases. From the perspective of proteins, the problem is then likely due to the small overall free energy for protein stability from non-covalent interactions, as well as tiny signal (length) difference between conformational change from each protein (un)folding step. For example, the hydrogen bond and hydrophobic interaction for protein folding stability are ~10 kcal·mol^−1^ ^8^. Consequently, the required energy or force resolution for observing each multistep unfolding is small, and the best single-molecule instruments currently available are difficult to resolve it.

The pioneering (un)folding study of Ribonuclease A by Nobel laureate Anfinsen takes advantage of reductive denaturation of the stable S-S bonds inside the protein and provides the first picture of how protein folds and unfolds^9^. Recently, a series of S-S bond reduction experiments in protein I27 using single-molecule AFM (atomic force microscopy) were performed^10–12^. Here, the protein unfolding provides an unambiguous marker to identify single disulfide bond reduction event and many unique S-S bond properties are revealed. Inspired by these works, we chose metalloprotein with relatively stable and multiple metal-ligand coordination bonds, to demonstrate the multistep unfolding phenomena of protein. Compared with individual non-covalent interaction, a metal-ligand coordination bond is of higher energy. We propose that the rupture of these stable metal-ligand bonds during protein unfolding pathways can be observed individually by a current single-molecule method such as AFM-based SMFS. Furthermore, the unfolding mechanism of metalloprotein is of high interest itself considering one more layer of complexity is added.

Metalloprotein is a ubiquitous type of protein in which metal ions are bound to the protein structure. The incorporation of metal ions significantly expands the functionality and stability of the protein^13–15^. In particular, folding of some proteins can only be achieved by metal ion binding with corresponding metal-ligand coordination bond formation. Consequently, the rupture scenario of these chemical bonds can be used to describe protein unfolding pathways and mechanisms. Thus, we chose such a metal-induced folding protein, metallothionein, to demonstrate.

**Figure 1:**
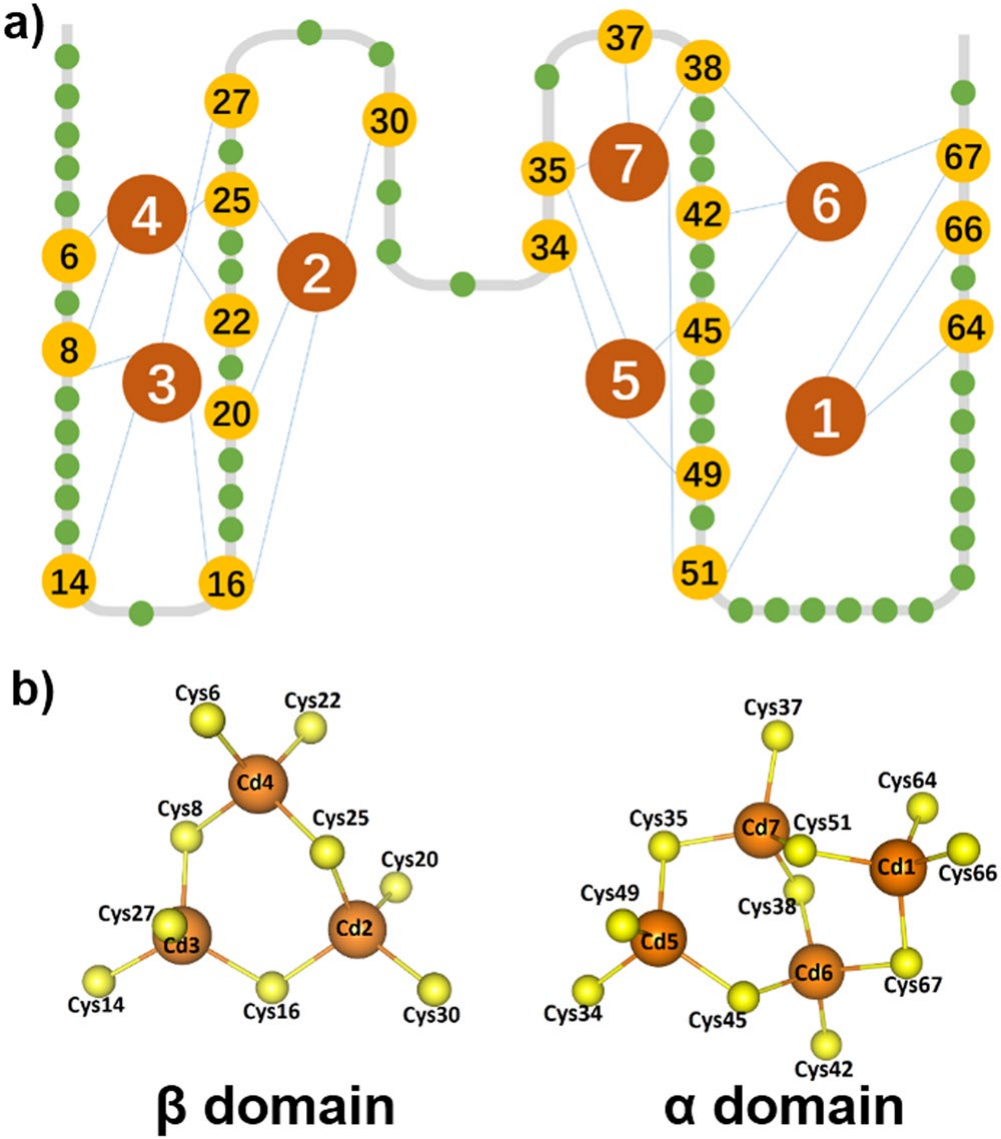
Schematics of Cd-MT and Cd-S clusters. (**a**) The cartoon of 2D MT structure shows an N-terminal β domain and a C-terminal β domain (PDB code: 2F5H) with seven cadmium ion and twenty cysteines, which are highlighted and numbered. (**b**) The schematics show the 3D structure of two metal clusters, Cd_3_S_9_ in the β domain and M_4_S_11_ in the β domain, respectively.

Human metallothionein III (MT) is chosen for our study. It is a small and cysteine-rich metalloprotein, which consists of 68 amino acids including 20 cysteines. As a result, it is a powerful metal binding protein which uses cysteines for metal coordination^16–19^. Intensive investigations using classic ensemble studies such as metal competing titration and mass spectroscopy have been performed to identify the metal association and dissociation sequence, and the metal binding sequence and preference^20–23^. It showed the metal could be added into the protein in a multistep fashion. For example, it binds to seven cadmium ions in its two domains, and the interaction between cadmium ions and the cysteinyl side chains leads to the formation of one Cd_3_S_9_ cluster and one Cd_4_S_11_ cluster that serve as the core of the β domain and α domain, respectively (Fig. 1a,b). Interestingly, it does not show an ordered secondary structure when no metal ion is bound. Thus, it naturally eliminates the most contribution of non-covalent interactions for protein structure and stability. Consequently, these Cd-S bonds determine the 3D conformation of MT, and the rupture scenario of the metal clusters equals the MT unfolding.

Here, we combined AFM-based SMFS and protein engineering techniques to investigate the β domain of MT unfolding process, and focused on the multistep (>2) unfolding scenario from the rupture of individual metal-ligand coordination bonds^24–28^. SMFS, including optical tweezer, magnetic tweezer, and single molecule-AFM, is a powerful technique to manipulate bio-macromolecule and characterize their nanomechanical properties^29–34^. Among them, single-molecule AFM has been widely applied to study protein folding/unfolding processes^35–39^ and its high force range enables direct manipulation and accurate measurement of stable chemical bond strength, including Au-S bonds and Si-O bonds^40–49^. Recently, it has been applied to the study of metal-ligand coordination bond strength, chemical reactivity and the interplay between the metal center and the protein structure in metalloprotein systems^50–53^. Thus, we chose it to detect multistep rupture scenarios of Cd_3_S_9_, which corresponds to metallothionein unfolding.

## Material and Method

### Protein engineering

Metallothionein used in this experiment was human metallothionein III. The gene encoding for MT was ordered from Genscript Inc. The gene of polyprotein (GB1)_3_-βMT-(GB1)_3_ was constructed in expression vector pQE80L using standard molecular biology techniques, where the GB1 (the B1 immunoglobulin-binding domain of *streptococcal* protein G, PDB code: 1PGA) was used as the marker domain for the single-molecule AFM experiments. The polyprotein was overexpressed in *Escherichia coli* strain BL21(DE3) with IPTG induction method, and the Cd ion was added by Schlenk techniques under an anaerobic condition. Further purification was performed using the gel filtration column Superdex 200 increase and the ion exchange column Mono Q5/50 using an FPLC machine (GE healthcare).

After purification, the concentration of the sample was adjusted to ~10 μM using the centrifugal filters. Also, the UV absorbance was detected for Cd-βMT by the Nanodrop 2000 spectrophotometer. The characteristic UV absorbance for the Cd-form proteins at 250 nm and 280 nm were recorded, respectively. Using the extinction coefficient of 8900 mM^−1^·cm^−1^ at 250 nm, the content of Cd was first calculated based on the UV-Vis spectrum, and the protein concentration was estimated by measuring the absorption at 280 nm. The accurate concentration was measured using the standard DTNB method based on the number of cysteine in the protein (DTNB, 8 M urea and 20 μM EDTA), by Nanodrop 2000 spectrophotometer. The exact number of cadmium ions in each protein was analyzed by inductively coupled plasma-mass spectrometry (ICP-MS) at Center of Modern Analysis, Nanjing University. Optima 5300DV ICP-MS from PerkinElmer was used.

### Single-molecule AFM experiments

Experiments were performed using a ForceRobot300 (JPK) atomic force microscope. The MLCT cantilever (Bruker Corp.) was calibrated in the solution using the equipartition theorem, and a spring constant of ~50 pN·nm^−1^ was usually obtained. All experiments were performed at room temperature in a Tris buffer of pH 7.4. The protein solution was first absorbed onto a glass coverslip and then was subjected to experiments after 10 minutes of incubation. The tip contacts the surface for hundreds of milliseconds and then retracts at a constant velocity. The pulling velocities for experiments were 400 nm·s^−1^ and 1000 nm·s^−1^, respectively. To ensure the data is from the real multistep unfolding events of βMT, not due to the background noise, only force-extension curves with stepwise rupture peaks whose cumulative ΔLc is ~8 nm (between 7 nm to 10 nm) were selected for further analysis. At least the ΔLc of one force peak is larger than 3 nm. Statistically, ~50 out of 1000 (~5%, 400 nm·s^−1^ condition) βMT unfolding curve showed such multistep unfolding events.

## Results

**Figure 2:**
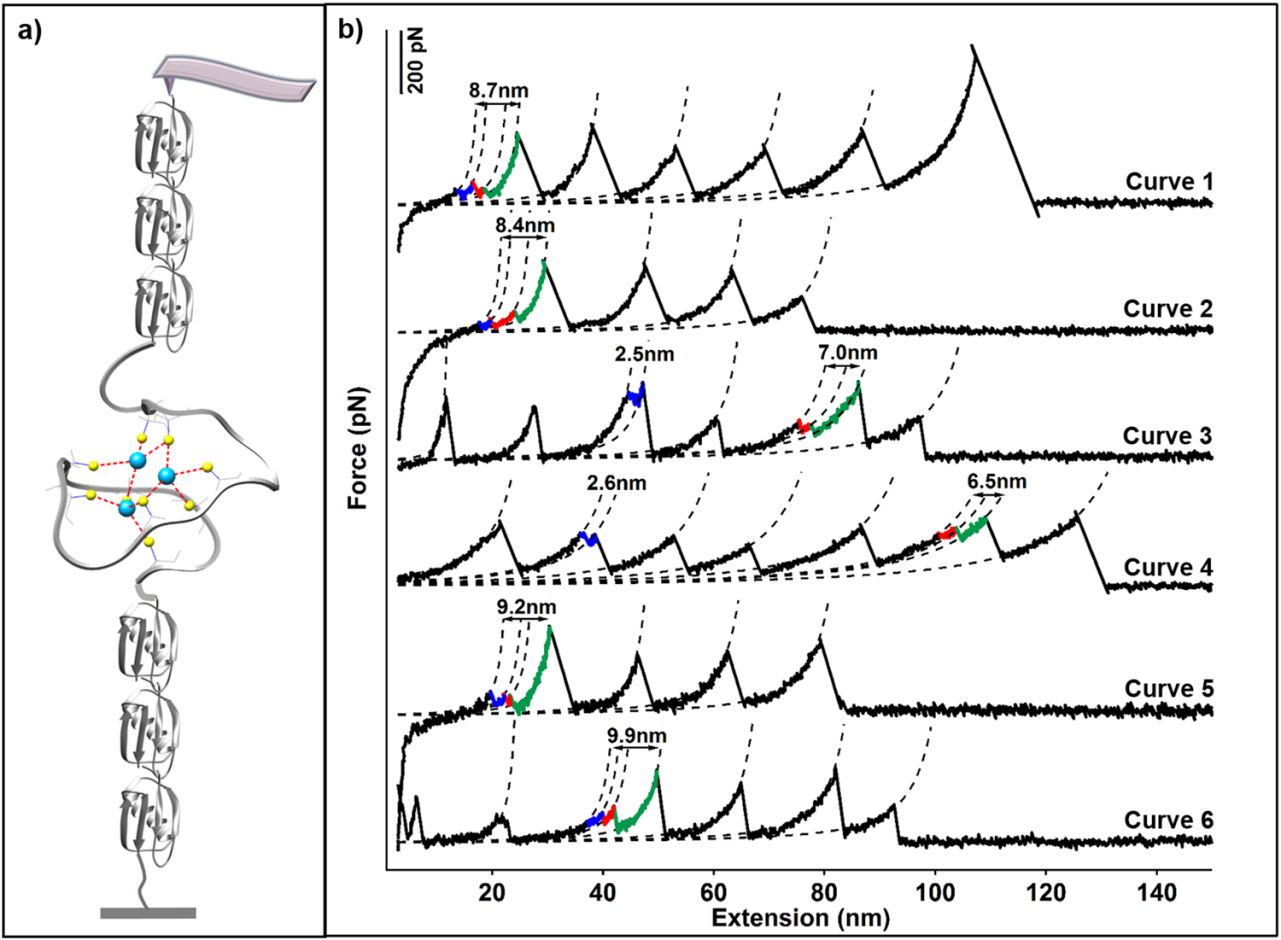
The three-step unfolding scenarios of βMT from the rupture of the M_3_S_9_ cluster revealed by single-molecule AFM. (**a**) The setup of single molecule AFM unfolding experiment for (GB1)_3_-βMT-(GB1)_3_. The Cd-form βMT is stretched mechanically between an AFM tip and a sample-deposited coverslip. The protein is unfolded under mechanical manipulation, as the rupture of the metal cluster. (**b**) Representative force-extension curve of the polyprotein (GB1)_3_-βMT-(GB1)_3_ showed sawtooth-like unfolding force peaks. The cumulative ΔLc value of the three peaks is of ~9 nm, which corresponds to the theoretical ΔLc of βMT unfolding (0.36 nm/aa * 25 aa–1.2 nm = 7.9 nm).

To study the unfolding mechanism of βMT with an M_3_S_9_ metal cluster, we constructed a (GB1)_3_-βMT-(GB1)_3_ polyprotein molecule and used Cd-βMT with a Cd_3_S_9_ for single-molecule AFM experiment (Fig. 2a). Here, βMT was sandwiched by a well-characterized marker protein GB1 with a known contour length increment (ΔLc) of ~18 nm^54^. The tip of AFM cantilever pressed onto the glass coverslip in which the protein solution was deposited, and the protein was captured between the tip and the coverslip through a non-specific interaction. By moving the cantilever at a constant pulling speed such as 400 nm·s^−1^, the protein was stretched and subjected to force and unfolded at last. The force-extension curve showed a typical sawtooth-like pattern in which each force peak corresponds to a specific protein or protein segment unfolding event (Fig. 2a). By fitting the curve elasticity with a worm-like chain (WLC) model which describes the function between applied force and polymer extension, multiple unfolding steps (>2) with the combination ΔLc of ~9 nm can be detected, besides GB1 unfolding events (Fig. 2b). Based on the protein structure, 25 amino acids (aa) are present between the first Cd_4_-SCys_6_ bond and the last Cd_2_-SCys_30_ bond in the Cd_3_S_9_ cluster^55^. Thus, the rupture of all Cd-S bonds in the β domain leads to the extension of 25 aa with a theoretical ΔLc value of 7.9 nm (0.36 nm/aa * 25 aa–1.2 nm). Consequently, the experimental unfolding result of βMT agrees well with the theoretical value of the complete unfolding of the individual β domain. Our results indicate that we observed multistep protein unfolding scenarios from the rupture of the Cd_3_S_9_ using single-molecule AFM.

**Figure 3:**
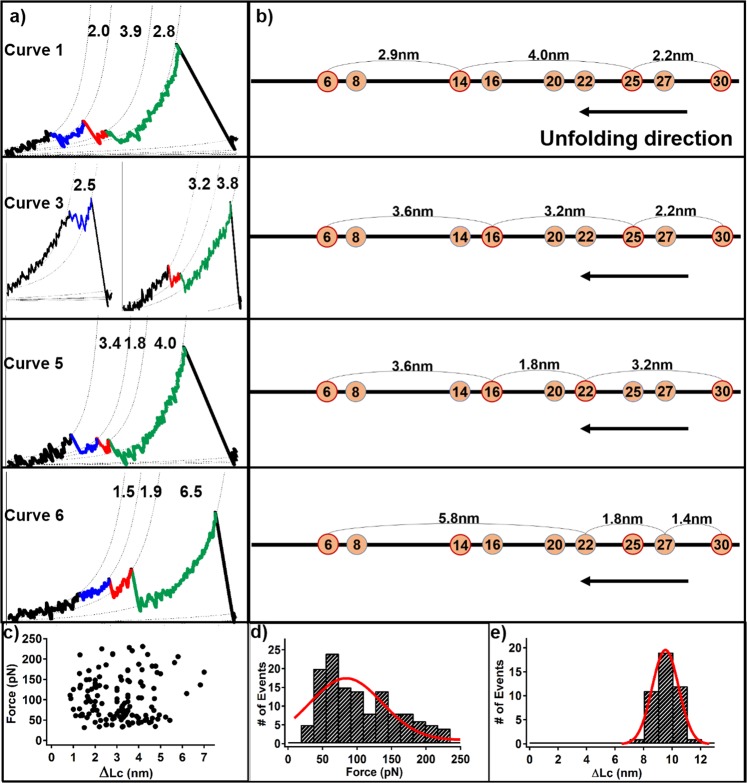
The three-step unfolding scenarios of βMT. (**a**) The local zoom of the representative curves in Fig. 2. The ΔLc combination is 2.0 + 3.9 + 2.8 nm for curve 1; 2.5 + 3.2 + 3.8 nm for curve 3; 3.4 + 1.8 + 4.0 nm for curve 5, and 1.5 + 1.9 + 6.5 nm for curve 6 in fig. 2. (**b**) The schematics display the rupture mechanism and sequence of M_3_S_9_ with the theoretical ΔLc, corresponding to the left curves. (**c**) The scatter plot describes the relationship between the unfolding force and ΔLc. (**d**) The force histogram of all individual three-step unfolding force peak showed an average rupture force of 105 ± 57 pN. (**e**) The histogram of the cumulative ΔLc of force peaks in each three-step βMT unfolding scenario shows a length value of 9.4 ± 0.9 nm.

Next, we focused on these complex three-step unfolding scenarios from the Cd_3_S_9_ metal cluster. At least four types of three-step unfolding events are distinguished and identified. The ΔLc combination of 2.0 + 3.9 + 2.8 nm was observed most frequently (Fig. 2b, curve 1 & 2, n = 16). Based on the topology of the protein and the metal cluster in the protein, this event can be possibly attributed to the rupture of the Cd_2_-SCys_30_ bond first, followed by the rupture of Cd_4_-SCys_25_ bond, and ended by the rupture of Cd_3_-SCys_14_ or Cd_4_-SCys_6_ bond_,_ corresponding to a theoretical ΔLc combination of 2.2 + 4.0 + 2.9 nm (Fig. 3a,b, curve 1 & 2). Three more different types of three-step unfolding events with ΔLc combinations of 2.5 + 3.2 + 3.8 nm (Fig. 2b, curve 3 & 4, n = 10); 3.4 + 1.8 + 4.0 nm (curve 5, n = 7) and 1.5 + 1.9 + 6.5 nm (curve 6, n = 11) were also detected, which can be explained from the rupture of specific Cd-S bonds. For example, the first event with ΔLc combination of 2.5 + 3.2 + 3.8 nm is attributed to the rupture of the Cd_2_-SCys_30_ bond first, followed by the Cd_4_-SCys_25_ bond, ended by the Cd_3_-SCys_16_ bond or the Cd_4_-SCys_6_ bond (theoretical ΔLc: 2.2 + 3.2 + 3.6 nm). More descriptions of these events can be found in Table 1. The relationship between force and ΔLc for all these three-step unfolding events is shown in Fig. 3c. The histogram of their unfolding forces showed a large distribution, which was between 30 pN and 240 pN, with an average force and standard deviation of 105 ± 57 pN (Fig. 3d). This large force distribution implies a quite small Δx from the rupture of the metal cluster, which is typical for metal-ligand bond. It is noted that only force-extension curves with the cumulative ΔLc of ~9 nm (9.4 ± 0.9 nm, n = 44) from the multistep rupture peaks are selected for analysis (Fig. 3e).

**Table 1:** Single-molecule AFM results for the three-step unfolding of Cd-βMT.

Theoretical ΔLc (nm)	Curve No.	Rupture Modes (The final step is from the rupture of one of the last two bonds)				Num.
2.2 + 4.0 + 2.9	1 & 2	Cys30	Cys25	Cys14	Cys6	16
2.2 + 3.2 + 3.6	3 & 4	Cys30	Cys25	Cys16	Cys6	10
3.2 + 1.8 + 3.6	5	Cys30	Cys22	Cys16	Cys6	7
1.4 + 1.8 + 5.8	6	Cys30	Cys27	Cys22	Cys6	11

To validate our observation of multistep unfolding scenario from βMT, we performed the single-molecule AFM unfolding experiments under a different and higher pulling speed, 1000 nm·s^−1^. As expected, most previous observed multistep unfolding scenarios were also observed, and the representative force-extension curves are shown in the Supplementary Information (Supplementary Fig. S1, n = 25). Similar as the protein unfolding process, the rupture force of the metal cluster is dependent on the pulling speed, as a higher rupture force (140 ± 66 pN, n = 75) was measured under higher pulling speed (Supplementary Fig. S2).

**Figure 4:**
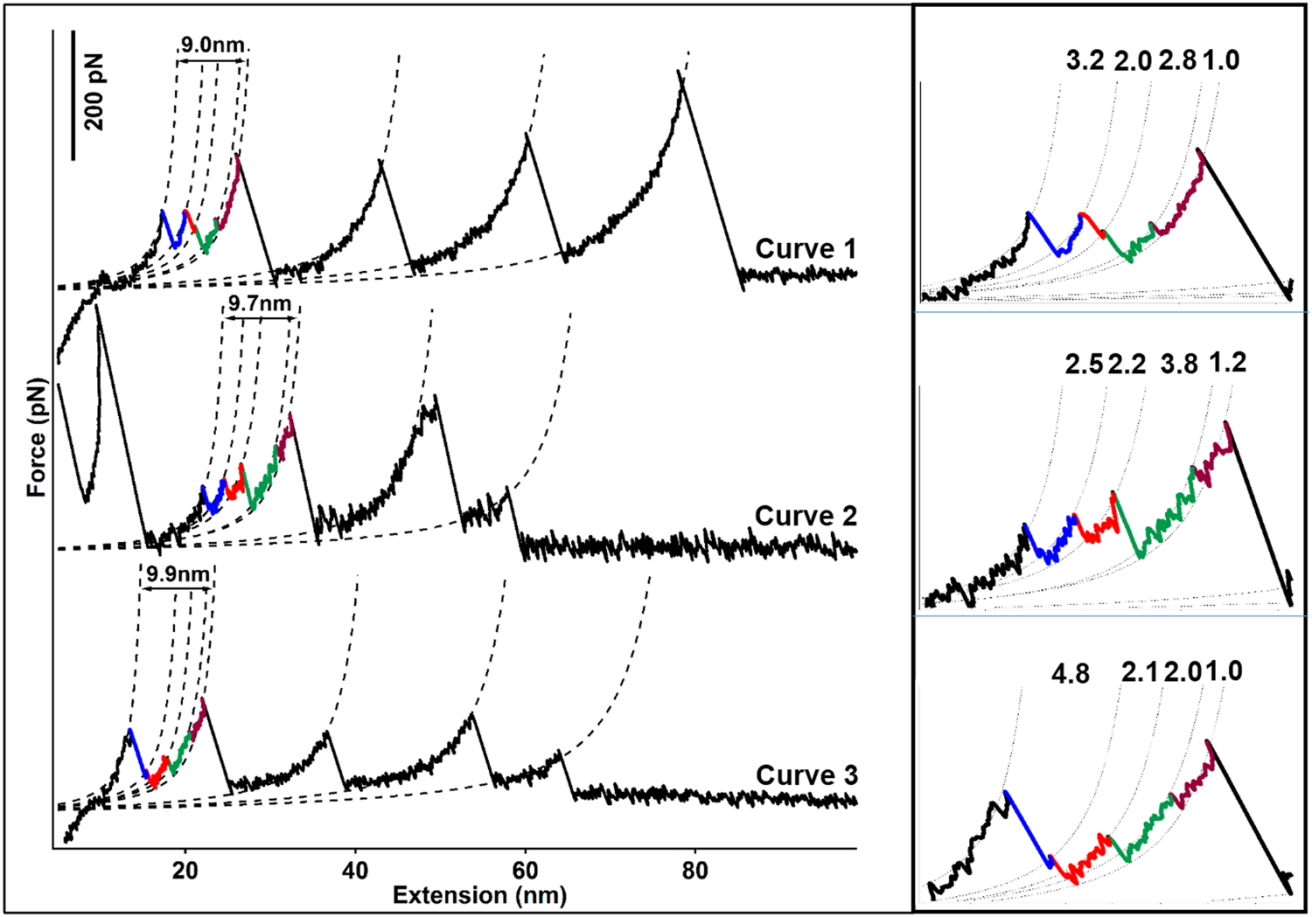
The unfolding scenario of βMT with four peaks. (**a**) Force-extension curves from the unfolding of (GB1)_3_-βMT-(GB1)_3_ with four peaks are shown: the different ΔLc combinations are: 3.2 + 2.0 + 2.8 + 1.0 nm for curve 1; 2.5 + 2.2 + 3.8 + 1.2 nm for curve 2, and 4.8 + 2.1 + 2.0 + 1.0 nm for curve 3. (**b**) The local zoom of the left curves shows the multiple unfolding peaks with specific ΔLc.

Moreover, besides the three-step unfolding scenario, we also detected even more complex unfolding scenario with four rupture peaks. Three different types of such unfolding scenarios of βMT with peaks of a shorter ΔLc were shown in Fig. 4. Their ΔLc combinations are: 3.2 + 2.0 + 2.8 + 1.0 nm (curve 1); 2.5 + 2.2 + 3.8 + 1.2 nm (curve 2), and 4.8 + 2.1 + 2.0 + 1.0 nm (curve 3). These unfolding events were from the rupture of the metal center in βMT based on the cumulative ΔLc value. However, the clear assignment of which Cd-SCys bond breaks is challenging. Nevertheless, they still demonstrate that a highly complex unfolding scenario of βMT is detected by using single-molecule AFM.

## Discussion

In this work, we detected several complex multistep unfolding scenarios of βMT from the rupture of a Cd_3_S_9_ metal cluster by single-molecule AFM experiments. Thanks to the mechanostable Cd-S bond in the protein with a rupture force above 30 pN, at least four different three-step metal cluster rupture scenarios were identified, as well as the observation of unfolding events with four rupture peaks. The summed value of each ΔLc, which equals to ~9 nm, confirmed their origin from the Cd_3_S_9_ metal cluster. In addition, the measured specific ΔLc upon each step led to the possible identification of the location, combination, and sequence of the ruptured Cd-S bonds for the three-step rupture scenario. As a metal-induced folding protein, we were able to use the rupture mechanism of the metal cluster to represent the multistep unfolding mechanism of βMT.

Metallothionein-3 is a small two-domain protein with 68 residues, particularly for the β domain with only ~30 residues. Typically, intermediate (un)folding states are less frequently observed in small proteins. Here, we identified four three-step unfolding scenarios of the β domain at the single Cd-S bond level. Small protein/domain with less structural motif typically shows a two-state (un)folding process with only one (un)folding pathway. Larger proteins, like GFP with a complex β barrel structure and T4 lysozyme with multiple α helices, show such complex properties^56,57^. For example, the ~240 amino acid GFP unfolds through at least two intermediate states along one unfolding pathway, and the ~150 amino acid T4 lysozyme with two domains unfolds through distinct multiple unfolding pathways. A recent study on a simple protein csp shows such a complex pathway as the unfolding of each secondary structure^58^. Also, the unfolding study on a membrane protein using improved cantilever showed tens of intermediates^4^. Nevertheless, these multiple unfolding pathways usually correspond to an unfolding step of a secondary structure intermediate like α-helix or β-strand^59^. It is noted that the β domain of MT has only ~30 amino acids without any secondary structure. As a result, it is unique to observe a 30 amino acid-length protein domain unfolding through multiple steps.

From the perspective of protein size, MT is a small protein. From the perspective of protein structure, it is a complex one consisting of seven cadmium ions and twenty-eight Cd-S bonds. As a metal-induced folding protein, the reason for such behavior can only be attributed to its three metal ions in the complex Cd_3_S_9_ cluster. We observed rupture events both from a single Cd-S bond as well as multiple Cd-S bonds during the domain unfolding. Nevertheless, the unfolding event from the rupture of multiple Cd-S bonds is dominant.

In the mechanical unfolding experiment, the protein was stretched from its N and C terminus and thus the force functioning as a denaturant was applied to the protein starting from these two points and transmitted to the protein backbone. The Cd-S bonds with the bound cysteines close to the N, C terminus should be subjected to force first than that of in the center. Consequently, a stepwise sequential Cd-S bond rupture scenario is more reasonable as the individual Cd-S bonds rupture one by one based on their location along the protein backbone. For example, the unzipping of β hairpin in protein usually results in a stepwise hydrogen bond rupture process with a smaller rupture force. Similar phenomena are widely observed for the unzipping of DNA and RNA harping molecule. In comparison, the stretch of β sheet under a shear pulling geometry results in a single-step unfolding with a much higher unfolding force. Here, hydrogen bonds behave like a bond network and thus are subject to force simultaneously. In the β domain of MT with Cd-S bonds, each Cd-S bond is relatively strong. Thus, it is reasonable to observe the sequential rupture of each Cd-S bond. However, the observation of a single rupture event from multiple bonds rupture is difficult to explain. Inspired by these hydrogen bond network features, we propose the M_3_S_9_ metal cluster can also be treated as a metal-ligand bond-based network in which mechanical force can be transmitted through and functions on other metal-ligand bonds and metal ions simultaneously. Thus, the central metal ion functions as a bridge, not only connecting to other Cd-S bonds and protein residues structurally but also transmitting the force/influence to other metal-ligand bonds. Consequently, several Cd-S bonds can be ruptured simultaneously.

In conclusion, using the β domain of metallothionein-3 with a complex metal cluster, we successfully detected the multistep unfolding scenario for a small protein. The multistep unfolding event is attributed to the stepwise rupture of the Cd_3_S_9_ metal cluster, which can be explained based on the different rupture sequences and combinations of specific Cd-S bonds.

## Supplementary information

supplementary information

## Data Availability

The datasets generated during and/or analysed during the current study are available from the corresponding author on reasonable request.
