# Supplementary material for: Multistep Protein Unfolding Scenarios from the Rupture of a Complex Metal Cluster Cd3S9
Source: Sci Rep. 2019 Jul 19;9:10518. doi: 10.1038/s41598-019-47004-y (PMC6642161; doi:10.1038/s41598-019-47004-y)
Supplement: Supplementary file 1 — supplementary information [file 41598_2019_47004_MOESM1_ESM.docx]

**Supplementary Information**

Multistep Protein Unfolding Scenarios from the Rupture of a Complex Metal Cluster Cd_3_S_9_

Guodong Yuan, Qun Ma, Tao Wu, Mengdi Wang, Xi Li, Jinglin Zuo, and Peng Zheng*

State Key Laboratory of Coordination Chemistry, School of Chemistry and Chemical Engineering, Nanjing University, Nanjing, Jiangsu, 21002, China

**The amino acid sequence of the βMT**

**(GB1)_3_-βMT-(GB1)_3_:**

HHHHHH-GS-

MDTYKLILNGKTLKGETTTEAVDAATAEKVFKQYANDNGVDGEWTYDDATKTFTVTE-RS-

MDTYKLILNGKTLKGETTTEAVDAATAEKVFKQYANDNGVDGEWTYDDATKTFTVTE-RS-

MDTYKLILNGKTLKGETTTEAVDAATAEKVFKQYANDNGVDGEWTYDDATKTFTVTE-RS-

**MDPETCPCPSGGSCTCADSCKCEGCKCTSCK**-RS-

MDTYKLILNGKTLKGETTTEAVDAATAEKVFKQYANDNGVDGEWTYDDATKTFTVTE-RS-

MDTYKLILNGKTLKGETTTEAVDAATAEKVFKQYANDNGVDGEWTYDDATKTFTVTE-RS-

MDTYKLILNGKTLKGETTTEAVDAATAEKVFKQYANDNGVDGEWTYDDATKTFTVTE

**Supplementary Figures**

**
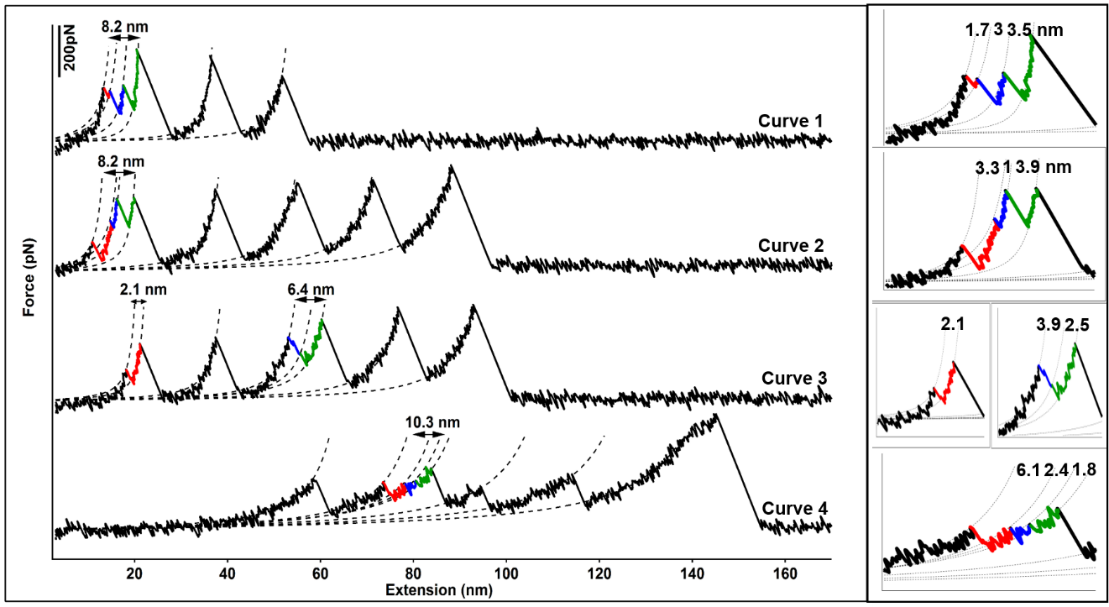
**

**Figure S1.** Representative force-extension curves of (GB1)_3_-βMT-(GB1)_3_ show the four different three-step unfolding scenarios from βMT, which were observed under the pulling speed of 400 nm·s^-1^. The number of detection for each scenario from curve 1 to 4 is 7, 2, 10, and 6, respectively. The right panel shows the local zoom of the left curve with the multiple unfolding peaks.


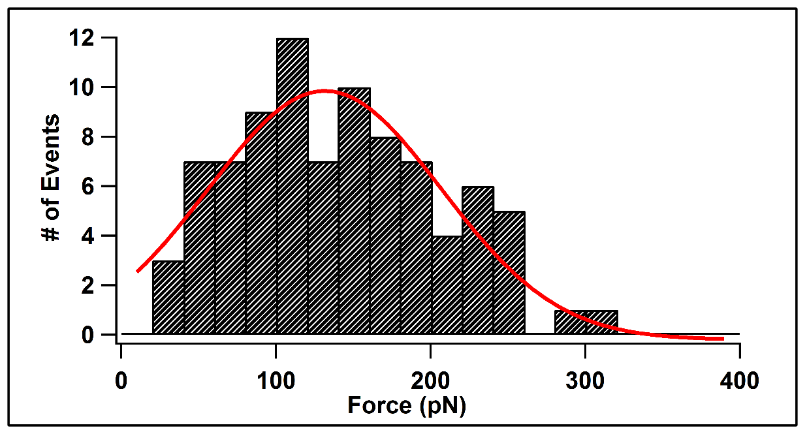


**Figure S2.** The unfolding force histogram for all the three-step unfolding force peaks under the pulling speed of 1000 nm·s^-1^ showed an average rupture force of 140±66 pN, n=75.
